# Luting of ceramic crowns with a self-adhesive cement: Effect of contamination on marginal adaptation and fracture strength

**DOI:** 10.4317/medoral.18544

**Published:** 2013-05-31

**Authors:** Slavena Slavcheva, Ivo Krejci, Tissiana Bortolotto

**Affiliations:** 1 Dr Med Dent: Visiting Assistant, Division of Cariology and Endodontics, School of Dental Medicine, University of Geneva, Switzerland; 2 Dr Med Dent, PD: Professor and Chairman, Division of Cariology and Endodontics, School of Dental Medicine, University of Geneva, Switzerland; 3 Dr Med Dent, MSC, PhD: Senior Assistant and Head of Research Laboratory, Division of Cariology and Endodontics, School of Dental Medicine, University of Geneva, Switzerland

## Abstract

Objectives: This study evaluated the percentages of continuous margins (%CM) and fracture strength (FS) of crowns made out from blocs of leucite-reinforced ceramic (IPS Empress CAD) and luted with a representative self-adhesive cement (RelyX Unicem) under four contaminating agents: saliva, water, blood, a haemostatic solution containing aluminium chloride (pH= 0.8) and a control group with no contamination. Study Design: %CM at both tooth-cement (TC) and cement-crown (CC) interfaces were determined before and after a fatigue test consisting of 600’000 chewing loads and 1’500 temperature cycles changing from 5º C to 50º C. Load to fracture was recorded on fatigued specimens. Kruskal-Wallis test was used to compare %CM and FS between the five groups with a level of confidence of 95%. Results: At the TC interface, no significant differences in marginal adaptation before loading could be detected between groups. After loading, a significant marginal degradation was observed in the group contaminated with aluminium chloride (52 ± 22 %CM) in respect to the other groups. No significant differences in %CM could be detected between the groups contaminated with saliva, water, blood and the control. At the CC interface, no significant differences in marginal adaptation were observed between the groups. The FS on loaded specimens was around 1637N, with no significant differences between groups as well. Conclusions: An adverse interaction of the highly acidic haemostatic agent with either dentin or the self-adhesive cement could explain the specimens’ marginal degradation. The self-adhesive cement tested in this study was no sensitive to moisture contamination either with saliva, water or blood.

** Key words:**Marginal adaptation, RelyX Unicem, contamination, all-ceramic crowns.

## Introduction

Resin-based cements are currently used for the luting of all types of indirect restorations, including all ceramic crowns (ACC), due to their improved mechanical properties, bond strength and higher aesthetics compared to conventional luting agents like zinc phosphate, glass ionomer and polycarboxylate cements (1,2). However, luting resin-cements that require the use of adhesive systems involve several steps of application such as etching, priming and bonding that can render them technique sensitive (1,3-5). One of the most investigated self-adhesive cements and the first of this category to be launched to the dental market in 2002 is RelyX Unicem (3M ESPE, Seefeld, Germany). The main characteristic of this material is that no pretreatment of the tooth surface is required (5).

Several studies have evaluated the mechanical and chemical properties (4,5-7), bond strength (2,5,6,8), interfacial characteristics (3,9) and sealing ability of this cement (10-12). The effect of surface contamination due to provisional cements on bond strength has also been assessed in previous studies (13,14). However, there is no information available on the effect of different contaminating factors on the marginal adaptation and fracture strength of ACC luted with self-adhesive cements. In particular, retraction cords used for gingival retraction usually contain astringent agents like aluminium chloride. It is known from previous studies that hemostatic solutions, due to their high acidity, can interfere with the bonding mechanism of self-etching adhesives (15). However, no information is available on the effect of these solutions when applied over dentin previous to the application of this type of luting material.

Therefore, the aim of the present study was to investigate how and to what extent different contaminating agents influence the sealing ability of a self-adhesive cement used for luting ACC. The null hypotheses tested were that there is no detrimental effect of contamination conditions (saliva, blood, water and aluminium chloride) on 1. The marginal adaptation before and after thermo mechanical loading and on 2. Fracture strength of previously fatigued ACC luted with RelyX Unicem.

## Material and Methods

Forty sound extracted human molars were selected for the study. Calculus and residual periodontal tissues were removed with a scaler and the teeth were cleaned with pumice powder. Subsequently they were stored in 0.1 % thymol solution until use. In order to place the teeth in the fatigue machine they were mounted with their long axes positioned vertically on custom made specimen holders using an autopolymerizing acrylic resin (Technovit 4071, Heraeus Kulzer GmbH, Wehrheim, Germany). They were randomly divided into five groups (four test and one control group, n=8). Details of each experimental group are given in  Table 1.

**Table 1:**
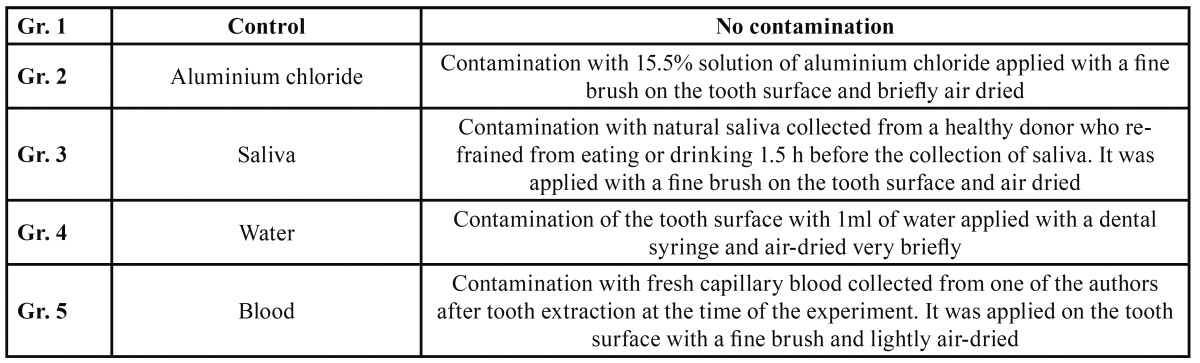
Description of the experimental groups.

Crown preparation was performed with the following dimensions: 1.5-2 mm reduction of the occlusal surface followed by a circular 1.2 mm wide shoulder prepared with a 80 µm grain-size cylindrical diamond bur (FG 8614, Intensiv, Grancia, Switzerland) under continuous water spray. The angle of convergence of the walls was 4-6 degrees and the approximate height of the final abutment was 5-6 mm. Finishing of the crown preparations was performed with a diamond bur with a grain size of 25 µm (FG 3526, Intensiv). The crowns were fabricated using a CAD/CAM system (CEREC System, Software version 3.10, Sirona, Bensheim, Germany). Cavity preparations were firstly coated with a titanium oxide-based agent (Cerec Propellant and Cerec Powder, VITA Zahnfabrik, Bad Säckingen, Germany) and digital impressions of the cavities were procured with an intraoral camera. After the digital design, crowns were milled out from leucite-reinforced ceramic crowns (IPS Empress CAD for CEREC and inLab LTA2/C14, Ivoclar Vivadent, Schaan, Liechtenstein, batch number M47001). Preparation of the crowns’ internal surface was performed with 5% hydrofluoric acid (Ceramics Etch, VITA, Zahnfabrik, Bad Säckingen, Germany) for 60 sec, rinsed and air-dried. Then a silane agent (Monobond S, Ivoclar Vivadent, Schaan, Liechtenstein) was applied, left undisturbed for 60 sec and then air-dried. Before luting, contamination of the tooth surfaces was performed with a solution containing aluminium chloride (Racestyptine, Septodont, France), saliva, water and blood as detailed in  Table 1. A group with no contamination served as control. After the contamination procedures all crowns were luted with a self-adhesive cement (RelyX Unicem, 3M ESPE, Seefeld, Germany, batch number 379121). The crown was seated applying pressure of 40 g/mm², which is equivalent to a force of 30N and corresponds to a moderate crown seating force (4). The cement was allowed to self-cure for 2-3 minutes and then the excess was removed using a spatula. Photo polymerization was performed with a light-curing device (Demetron Demi LED, Kerr Corporation, CA, USA) operating at no less than 1,000 mW/cm² for 60s from occlusal, lingual, buccal, mesial and distal surfaces. Curing efficiency was periodically checked after each luting with a radiometer. After polymerization the margins were polished using flexible aluminium oxide discs of different grain sizes (Sof-Lex, 3M ESPE). The final polishing was checked using a binocular magnifying lens (Leica MZ6, Heerbrugg, Switzerland) under 2.0x magnification.

After polishing, the specimens were stored in tap water at 37 °C for one week; then the restored teeth were loaded in a computer-controlled chewing machine. Thermal and mechanical loading was applied simultaneously. Thermal cycling was carried out in running water with temperatures changing 1,500x and ranging from 5 °C to 50 °C with a cycle time of two minutes. The mechanical stress comprised a total of 600’000 load cycles transferred to the centre of the occlusal surface with a frequency of 1.7 Hz and a maximal load of 49 N. The load was applied using a natural lingual cusp taken from an extracted human molar tooth.

After completion of the polishing procedure, i.e. before loading, and after loading, the teeth were cleaned with rotating brushes and toothpaste. Then impressions with a polyvinylsiloxane impression material (President light body, Coltène Whaledent, Altstätten, Switzerland) were taken from the mesial and distal section of the crown margin of each restoration. Subsequently gold-coated epoxy resin replicas were prepared for quantitative margin analysis in a Scanning Electron Microscope (XL20, Philips, Eindhoven, The Netherlands) at 200x magnification. For the marginal evaluation a custom-made module programme with an image processing software (Scion Image, Scion Corp, Frederik, MA 21703, USA) was used. Percentages of continuous or perfect margins (%CM) were reported for the entire marginal length and were measured for interfaces tooth cement (TC) and crown cement (CC).

After the fatigue process, fracture resistance of the specimens was tested in a universal testing machine (Instron Model 1114, MA, USA). The teeth were loaded perpendicularly to the occlusal surface at a crosshead speed of 0.5 mm/min. A spherical steel ball of 2 mm² used to transmit the force. Load force continued until tooth fracture occurred, and fracture force was recorded in Newtons (N) (16).

The statistical analysis of the data was performed with SPSS 16.0 for Windows. Percentages of continuous margins (%CM) before and after loading (initial and terminal respectively) were reported for the five groups and for both TC and CC interfaces. Data on marginal adaptation and fracture strength was evaluated with Kruskal-Wallis and post-hoc test. The level of confidence was set to 95%.

## Results

The %CM for both examined interfaces before and after thermo mechanical loading are shown in  Table 2. Before loading and at the TC interface there was no statistically significant difference between groups (p=0.684). After loading the difference between groups was significant (p=0.002), the %CM being the lowest in the group contaminated with aluminium chloride. No significant differences were observed between the groups contaminated with saliva, water, blood and no contamination at all. At the CC interface there were significant differences before loading (p=0.048) and the lowest scores of marginal adaptation were observed in the groups contaminated with aluminium chloride and saliva. However, no differences between groups were observed after loading (p=0.620) and almost 100%CM were observed at this interface.

**Table 2:**
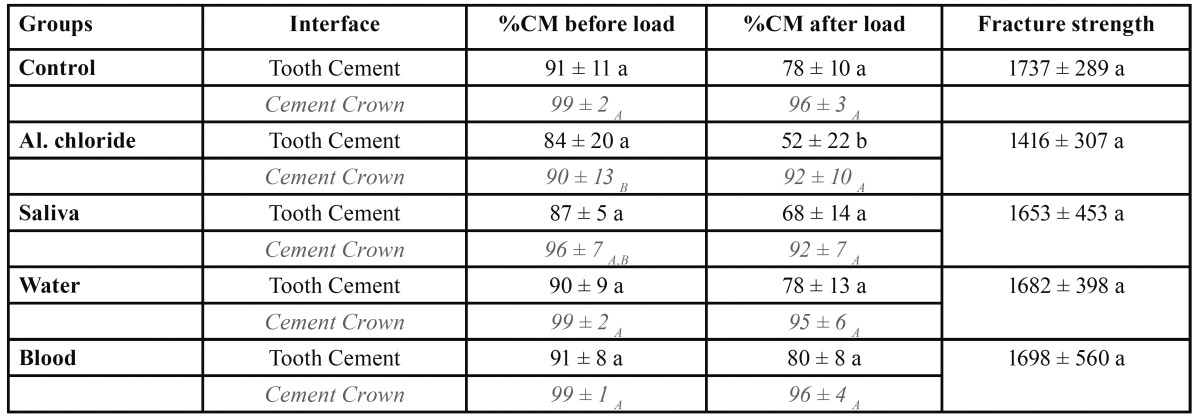
Results of marginal adaptation (Mean ± SD) expressed as percentages of continuous margins (%CM) and of fracture strength (N). Levels connected by different letters are significantly different and apply to each column; differences at the Cement Crown interface in upper case letters and differences at the Tooth Cement interface in lower case letters.

In terms of fracture strength, no statistically significant differences were observed between the experimental groups (p=0.558). Still, the lowest fracture load (1416 ± 307 N) was recorded for the group contaminated with the haemostatic agent.

## Discussion

Contamination of cavity margins with saliva, water, blood, plasma and gingival crevicular fluid is usual in restorative dentistry especially when the preparation margins are located subgingivally (8). Although an adhesive procedure should be avoided in such cases due to the risk of contamination resulted from the proximity to the gingival tissue, recent reports advocated the relocation of subgingivally located margins with resin composite buildups in order to situate them para or supragingivally (17,18). Another alternative would be to use an adhesive luting material that is tolerant to humidity. In this sense, even if self-adhesive cements are commonly used for the adhesive luting of indirect restorations, as their application procedure is simple, little is known about their tolerance to contaminating agents that can be present at the gingival area. As restoration margins are continuously exposed to the oral environment, early signs of degradation may be visible at this level. Therefore, the analysis of marginal adaptation before and after a fatigue process together with a fracture test would provide us with the information on the long-term resistance of the luting interface and the influence, or not, of this interface degradation on fracture load.

The results of the present study could show that while water, blood and saliva had no negative effect on the marginal adaptation, degradation was evident when the tooth surface was contaminated with the gingival retraction material containing aluminium chloride. Therefore, the 1st null hypothesis stating that there is no effect of contamination conditions on the marginal adaptation of ACC luted with RelyX had to be rejected. Two chemical mechanisms might be responsible for the adverse effect of aluminium chloride on the dentin/self-adhesive cement’s marginal interface. Firstly, most haemostatic solutions used for gingival retraction have low pH ranging from 0.7- 2.0. The solution containing aluminium chloride has a pH of 0.8. The prolonged contact of these solutions with dentin surface, especially in cases of subgingival margins, has been found to cause a removal of the dentinal smear layer and opening of the dentin tubuli (19). It is possible that the solution used for gingival retraction simply “etched” dentin. A poor infiltration of this dentin with the self-adhesive cement, which is highly viscous, could have contributed to the formation of marginal gaps. Secondly, the high acidity of the gingival retraction solution could inhibit the self-curing components of the resin cement (11) during the interval of 2-3 min used to enable self-curing. This chemical incompatibility has already been observed between highly acidic self-etch adhesives and chemical/dual cured composites also due to the acidity of the primer that inactivates the tertiary amines present in the resin composite (20).

The fact that no significant differences in marginal adaptation were observed when dentin surface was contaminated with the other agents (water, saliva and blood) can be explained by the chemical nature of the self-adhesive cement. RelyX combines glass ionomer, adhesive and composite technology (information from the manufacturer). It contains methacrylated phosphoric esters, which are necessary for the demineralization and bonding to dentin, that need water to be used as a mediator for their ionization (9). RelyX has two types of setting reactions: an acid-base reaction having as final products calcium phosphate and responsible for the chemical adhesion of the luting agent to the tooth structure, and a photo- and redoxinitiated polymerization reaction responsible for the micro-mechanical interlocking of the luting agent (9,21). It is known from previous studies that in the case of resin-modified glassionomer materials, a certain water flux exists within the maturing cement, depending on environmental moisture changes (22). Moreover, continuous water flux between the environment and the cement should occur until there is a balance of osmotic pressure, otherwise self-desiccation would occur within the cements’ mass (23). In the context of our study, environmental moisture changes due to contamination with saliva or blood did not seem to affect the marginal adaptation, justifying why similar results were observed after loading.

In respect to blood contamination, it has been advocated that blood could form a physical barrier on the tooth surface, interfering with the unset material (24). However, in the present study no negative effect was observed on marginal adaptation when dentin surface was contaminated with blood. One explanation to our results could be that to mimic the clinical situation as close as possible, fresh capillary blood obtained from a patient after tooth extraction was used for the experiment. As blood plasma is also composed in a high percentage by water, this might explain why the results in marginal adaptation did not differ from those obtained when contamination was performed with the other agents.

Finally, no differences in fracture resistance were found between the different groups, accepting the 2nd null hypothesis. This means that the ability to resist to crack propagation was independent of contamination. Loads to failure of around 1637N were recorded; these scores are in line with literature that used similar methodology for this self-adhesive cement but no contaminating agents (10). The fact that the cement-crown (CC) interface was almost intact (%CM near 100) after loading may have also contributed to the similar results of fracture load between groups. These high results of marginal adaptation were due to an optimal preparation of the restoration’s internal surface prior to luting. Preparation of the ceramic intaglios’ surface with hydrofluoric acid, silanisation and bonding agent, as performed in this study, has been found to efficiently prepare the restorations’ surface for bonding (25). The resin strengthening effect provided by bonding to porcelain has been previously explained by a bridging or crack healing effect by either strong bonding of the resin to the ceramic surface or the silane molecules entering the crack (26-28).

## Conclusions

Ceramic crowns luted with RelyX were not negatively affected by moisture contamination with saliva, water or blood in both marginal adaptation and fracture strength. However, retraction solutions based on aluminium chloride should be completely removed from the tooth surface before luting.
